# Femoral vein cutdown as a bailout in failed percutaneous retrieval of migrated coronary sinus reducer: images in cardiology

**DOI:** 10.1093/ehjcr/ytaf347

**Published:** 2025-07-21

**Authors:** Azhar Farooqui, F Aaysha Cader, Mohsin Farooq, Prashanth Raju

**Affiliations:** Department of Cardiology, Kettering General Hospital, Rothwell Road, Kettering NN16 8UZ UK; Department of Cardiology, Kettering General Hospital, Rothwell Road, Kettering NN16 8UZ UK; Department of Cardiology, Kettering General Hospital, Rothwell Road, Kettering NN16 8UZ UK; Department of Cardiology, Kettering General Hospital, Rothwell Road, Kettering NN16 8UZ UK

## Summary

Coronary sinus reducer (CSR) stent migration is a well-recognized procedural complication, which can occur at various stages of device implantation.^1^ We share a case of migrated stent retrieval using snare technique and femoral vein cutdown in a 70-year-old patient with history of ischaemic heart disease, prior coronary artery bypass procedure, refractory angina symptoms, inducible ischaemia on functional imaging, and no options for percutaneous revascularization.

## Case presentation

**Figure 1:**
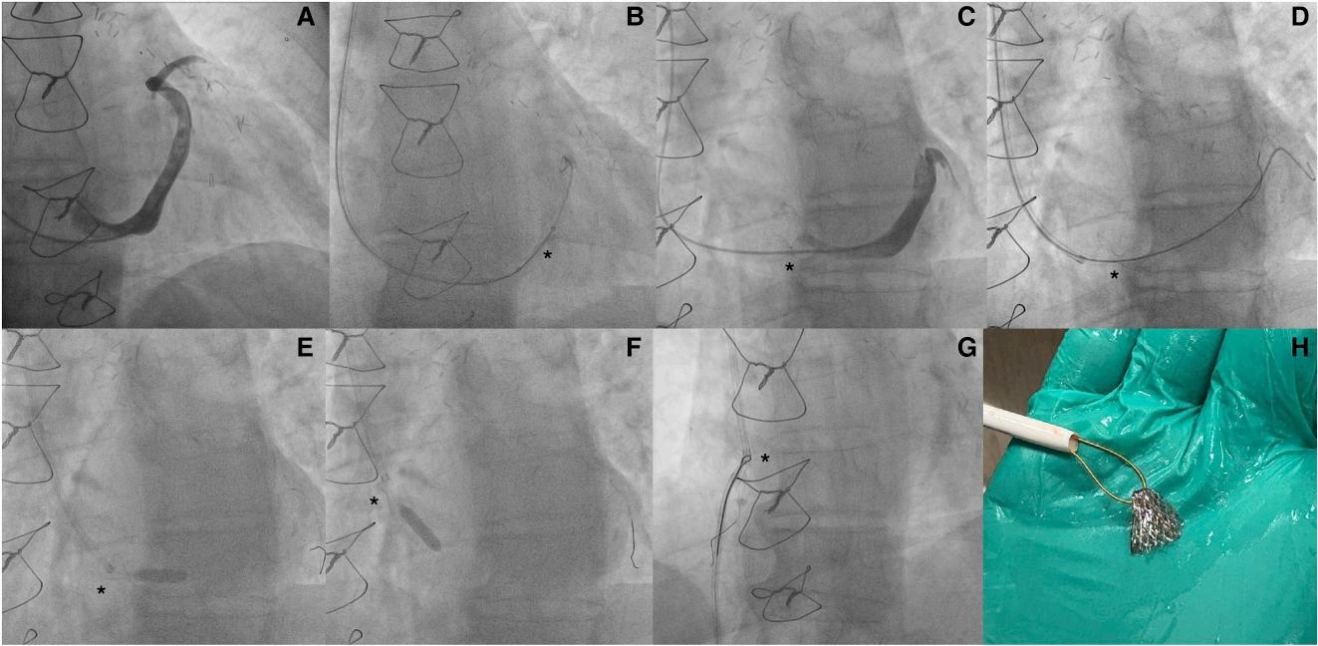
Coronary sinus angiogram (*A*); coronary sinus reducer-mounted balloon positioned in the mid-coronary sinus and inflated to 1 atm to anchor the stent onto the balloon (*B*); during deployment, the coronary sinus reducer stent migrated proximally towards the ostium of coronary sinus, and the neck and proximal portion of the stent was found to be within the right atrial cavity (*C*); a Sion Blue Extra support wire (Asahi Intecc USA, Inc.) was used to cross through the neck of the coronary sinus reducer stent into distal coronary sinus (*D*). A 5.0 × 15 mm TREK balloon (Abbott Vascular) was inserted and inflated distal to the coronary sinus reducer stent, to prevent further device migration (*E*). The secured coronary sinus reducer was then pulled into the right atrial cavity (*F*). A snare (eV3 Amplatz GOOSE NECK Snare Kit) was utilized with the lasso of the snare secured at the neck of the coronary sinus reducer stent (*G*). Patient underwent a right femoral vein cutdown under local anaesthesia with successful extraction of the coronary sinus reducer (*H*). * in the images marks the coronary sinus reducer in the image sequences.

Using a 5 Fr AL1 diagnostic catheter, a coronary sinus (CS) angiogram was obtained (*Figure 1A*). Coronary sinus reducer-mounted balloon was then positioned in the mid-CS and inflated to 1 atm to anchor the stent onto the balloon (*Figure 1B*), before repositioning it to proximal CS, further inflating to 4 atm, allowing the recommended 20% device oversizing relative to the CS cross-section. During deployment, the CSR stent migrated proximally towards the ostium of CS, and the neck and proximal portion of the stent was found to be within the right atrial cavity (*Figure 1C*; Supplementary material online, *Video S1*). A Sion Blue Extra support wire (Asahi Intecc USA, Inc.) was used to cross through the neck of the CSR stent into distal CS (*Figure 1D*; Supplementary material online, *Video S2*). A 5.0 × 15 mm TREK balloon (Abbott Vascular) was inserted and inflated distal to the CSR stent, to prevent further device migration (*Figure 1E*). The secured CSR was then pulled into the right atrial cavity (*Figure 1F*; Supplementary material online, *Video S3*). A right femoral venous access was established using an 11 Fr venous sheath (largest available to us at the time). A snare (eV3 Amplatz GOOSE NECK Snare Kit) was utilized. The lasso of the snare was secured at the neck of the CSR stent (*Figure 1G*; Supplementary material online, *Video S4*), and the CSR stent gradually retracted. Unfortunately, the CSR became perpendicularly aligned against the tip of the sheath, wedged and indenting the wall of the common femoral vein, without further collapse inside the sheath (see Supplementary material online, *Video S5*). Patient underwent a right femoral vein cutdown under local anaesthesia with successful extraction of the CSR (*Figure 1H*).

## Supplementary Material

ytaf347_Supplementary_Data

## Data Availability

All data are incorporated into the article.
